# Label-free detection of biomolecular interactions in real time with a nano-porous silicon-based detection method

**DOI:** 10.1186/1477-5956-6-31

**Published:** 2008-11-04

**Authors:** Martin Latterich, Jacques Corbeil

**Affiliations:** 1 Faculty of Pharmacy, University of Montreal, Montreal, QC, H3T 1J4, Canada; 2 Université Laval, Infectiology and Cancer Research Centers, 2705 Blvd. Laurier office T3-67, Quebec, QC, G1V 4G2, Canada; 3 Biomatrica, 5627 Oberlin Drive, San Diego, CA 92121, USA

## Abstract

**Background:**

We describe a biosensor platform for monitoring molecular interactions that is based on the combination of a defined nano-porous silicon surface, coupled to light interferometry. This platform allows the label-free detection of protein-protein and protein-DNA interactions in defined, as well as complex protein mixtures. The silicon surface can be functionalized to be compatible with traditional carboxyl immobilization chemistries, as well as with aldehyde-hydrazine bioconjugation chemistries.

**Results:**

We demonstrate the utility of the new platform in measuring protein-protein interactions of purified products in buffer, in complex mixtures, and in the presence of different organic solvent spikes, such as DMSO and DMF, as these are commonly used in screening chemical compound libraries.

**Conclusion:**

Nano-porous silicon, when combined with white light interferometry, is a powerful technique for the measurement of protein-protein interactions. In addition to studying the binary interactions of biomolecules in clean buffer systems, the newly developed surfaces are also suited for studying interactions in complex samples, such as plasma.

## 

Protein-protein and protein-DNA interactions are at the center of cellular regulation. Obtaining a large-scale quantitative assessment of protein binding would greatly assist in system biology modeling, diagnostics, and drug screening [1]. The sensitive and versatile detection of macromolecular interactions without the aid of fluorescent or luminescent labels is considered one key goal of proteomics [2]. The cost and potential interference of fluorescent protein tags or coupled fluorophores with the kinetic parameters of protein-protein and protein-DNA interactions have led to serious efforts to develop and optimize label-free detection of proteins. Several technologies have been developed for that purpose [3-6]. For example, surface plasmon resonance (SPR) performed on gold surfaces has been the mainstay of detecting interactions between protein pairs or protein ligand binding [7]. Recently, other technologies have been employed to measure by optical interferometry protein-protein interaction of proteins immobilized to the surface of optical fibers or planar waveguide surfaces [8-11]. The choice of substrates other than gold has the advantage, other than cost, that these surfaces can accommodate a wider variety of coupling chemistries, making them more versatile in terms of attaching a greater range of molecules.

This study reports on the use of a defined nano-porous silicon substrate coupled to reflected white-light interferometry to measure refractive index changes on the porous surface (Figure 1a). When protein is covalently coupled to the porous surface, or when protein binding to a receptor surface occurs, the change of refractive index conferred by the immobilized protein leads to a spectral shift, resulting in a wavelength shift of the spectrum. This wavelength shift, called interferogram (Figure 1b), is a measure of protein mass bound to the surface [12]. Using an industrial prototype of this technology, built and supplied by Silicon Kinetics, Inc. , we have been adopting the technology to allow the measurement of protein-protein interactions with surface immobilized receptor molecules. Specifically, incident white light is projected onto the porous silicon surface. Reflected light from the porous silicon/fluid interface and reflected light from the bulk silicon/porous silicon interface are focused onto a diffraction grating from where emitted light is measured by a photo detector. Shifts in spectral properties as a result of protein binding are computed and translated into an optical path difference (OPD) shift, which is proportional to the mass of surface bound protein. The binding of a ligand to the receptor molecule generated an OPD shift, demonstrating that the ligand indeed bound the receptor (Figure 2).

**Figure 1:**
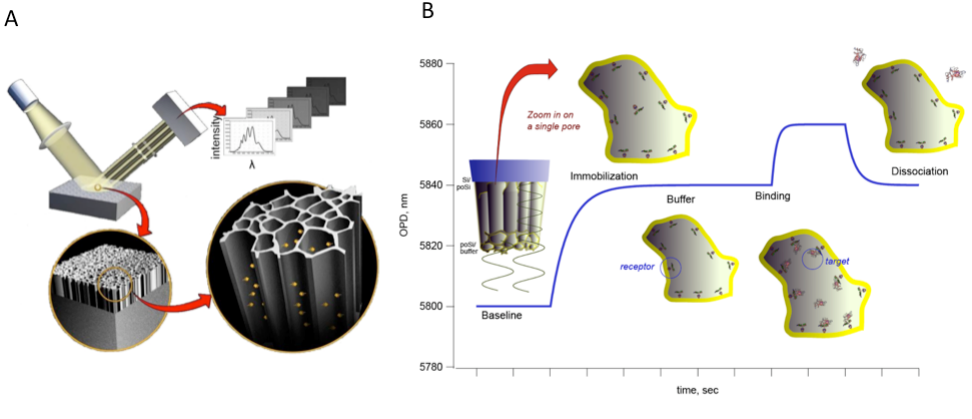
A) Artist's rendering of nano-porous biosensor principle. B) Schematic representation of interferogram of a typical nano-porous silicon biosensor experiment. (Source: Silicon Kinetics).

**Figure 2:**
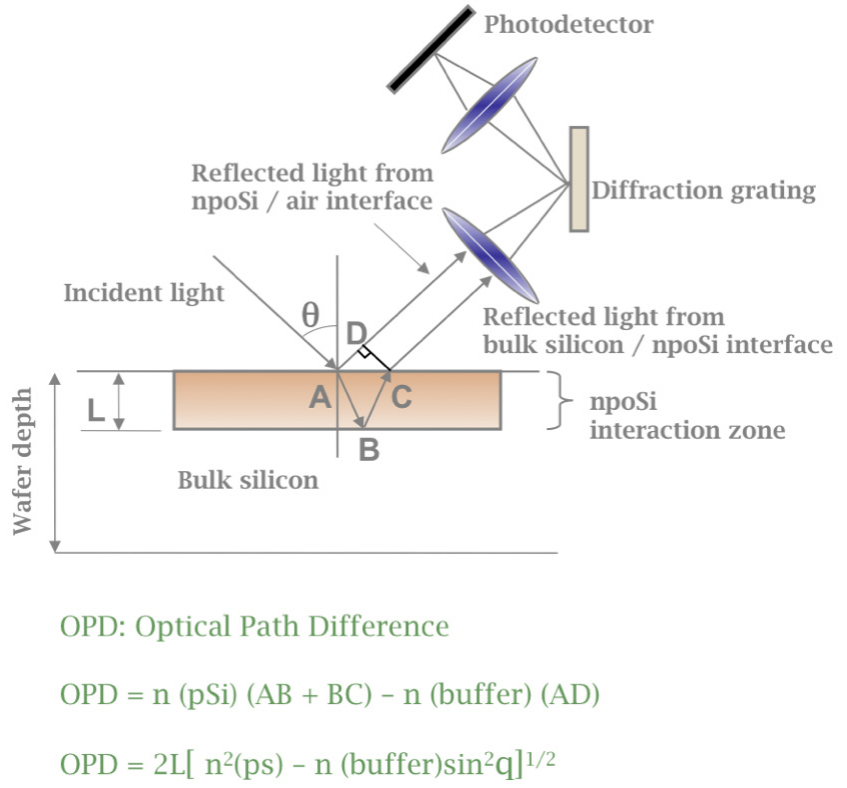
Overview of optical path and physic of signal acquisition (Source: Silicon Kinetics).

In brief, to generate amine-reactive porous silicon chips, an activation step with 1-ethyl-3-(3-dimethylaminopropyl) carbodiimide (EDC) and N-hydroxysulfosuccinimide (S-NHS) is required immediately before immobilizing protein (Figure 3). This approach has the advantage that the activated surface can react with amines donated by the unmodified protein to be immobilized, albeit the reaction occurs at lower efficiency when compared to other surface immobilization chemistries but serves as a standard method that can accommodate a large number of protein interaction pairs and groups. Proteins will then be presented to the activated surface, and will react with exposed amines with the NHS-moiety donated by the surface [13].

**Figure 3:**
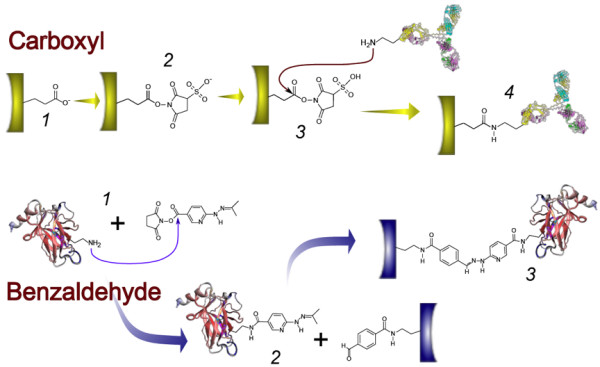
Nano-porous silicon immobilization chemistries. Top row: Carboxyl chemistry. Carboxyl functionalized nano-porous silicon chips (1) are activated and functionalized with EDC/sulfo-NHS. The sulfo-NHS ester functionalized surface (2) will react with amines contributed by the protein to be immobilized (3), leading to the formation of a stable amine bond between protein and surface (4). Bottom row: The protein to be immobilized is reacted with the bifunctional HyNic reagent (1), which will react with amines contributed from the protein to form hydrazine-functionalized protein. The hydrazine-functionalized protein is reacted with benzaldehyde functionalized nano-porous silicon surface (2), which will lead to a stable attachment of the protein to the surface through a hydrazone bond (3).

Benzaldehyde activated surfaces have the advantage that they are stable and can be manufactured in advance of the reaction with appropriately functionalized proteins (Figure 3). The immobilization chemistry is based on the efficient reaction between benzaldehyde and an aromatic hydrazine, which occurs at neutral pH and forms a stable hydrazone bond [14]. While proteins to be bound to a benzaldehyde surface need to be functionalized with hydrazone prior to their immobilization, thus making this method strictly speaking not label free, this step has the advantage that large batches of hydrazone functionalized protein can be prepared, resulting in greater reproducibility of immobilization as opposed to EDC/S-NHS ester based immobilization. Furthermore, separating the functionalization step from the immobilization step has the advantage that proteins labeled with a hydrazine functional group can be affinity or activity-purified, thus reducing the pool of molecules inactivated by the labeling reaction. Due to the more robust chemistry, the surface can be utilized at higher efficiency, resulting in higher labeling densities, hence increasing detection sensitivity. It is thus possible to label large batches of protein with a reagent that functionalizes the protein with an aromatic hydrazone, and to then aliquot and freeze these functionalized reagents. The reaction of a hydrazine formed from an aromatic hydrazone in aqueous solution will react with an aldehyde-functionalized surface after formation of a Schiff-base to form a stable, covalent hydrazone bond. Therefore, this reaction has many advantages; it occurs at neutral pH (and thus compatible with pH-labile proteins) and leads to higher surface packing densities that other receptor immobilization methods yielding better sensitivity for detection. Given the much lower variance in immobilization efficiency between different experiments, it is therefore suited for applications where large datasets need to be compared across different chips, or where a higher packing density is needed.

To test different surface immobilization chemistries, their efficiency of immobilization, stability, ability to interact with other proteins subsequent to immobilization, and non-specific binding properties, we resorted to the well-characterized interaction between streptavidin and biotinylated bovine serum albumin (BSA). Specifically, we immobilized streptavidin to carboxyl surfaces, or to benzaldehyde functionalized surfaces as outlined in the Materials and Methods section. Immobilized streptavidin was then exposed to BSA, biotinylated BSA, or 10% lipid-depleted rat plasma while recording OPD shift in real time. Succinimidyl 6-hydrazinonicotinate acetone hydrazone (HyNic)-labeled streptavidin efficiently and permanently reacts with the aldehyde of the porous silicon surface, as demonstrated by the stable baseline after washing. A typical experiment results in an OPD shift of 32 units at an estimated surface coverage of 40% (Figure 4). This streptavidin-coated surface can efficiently recruit biotinylated BSA from solution, until equilibrium is reached. The displacement is typically 13 units. Application of 10% (v/v) rat plasma does not yield any significant modulation in binding activity, suggesting that the benzaldehyde surface is well suited for applications where elimination of non-specific binding is paramount. To test the extent of non-specific binding at higher plasma concentration, we incubated a benzaldehyde surface with increasing amounts of rat plasma (Figure 5). With a low plasma concentration of 10% (v/v), we do not observe surface binding as measured by OPD shift, however, increased concentrations of 20 to 40% (v/v) do indeed show a low degree of temporary non specific binding during sample application. Surfaces exposed to such high plasma concentrations still remain competent for recruiting specific proteins, suggesting that even at plasma concentrations much higher than the commonly used 10% diluted plasma, are still compatible with the nano-porous silicon surfaces used in this study.

**Figure 4:**
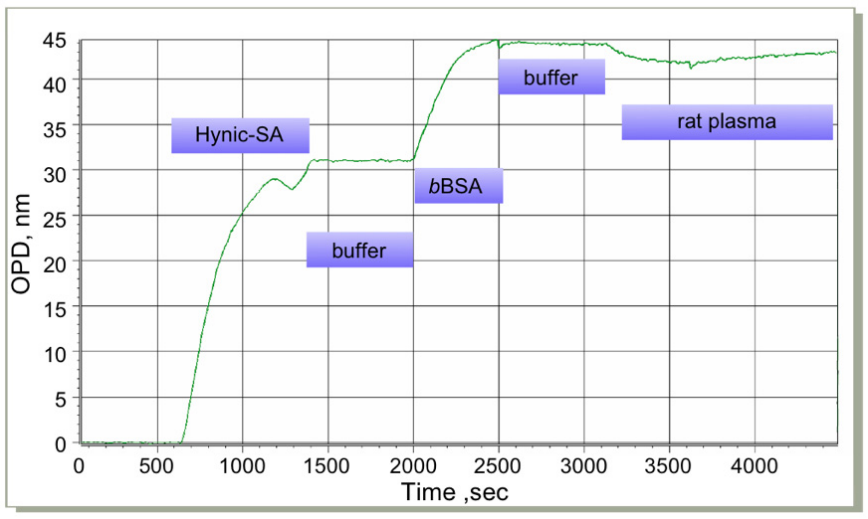
Typical full immobilization and binding run with non-specific binding test using a benzaldehyde biochip and hydrazone cross-linking chemistry. The signal shown is the result of subtracting the sample channel from the reference from the differential flow cells used with Ski Pro. 50 μL sample and reference volumes each were exposed to the sensor surface over 500 seconds. We applied 0.5 mg/mL HyNic-labeled streptavidin in the sample channel and conjugation buffer in the reference channel for 500 seconds, followed by 0.1 mg/mL biotinylated BSA. 10% delipidated rat plasma was run as a non-specific binding control.

**Figure 5:**
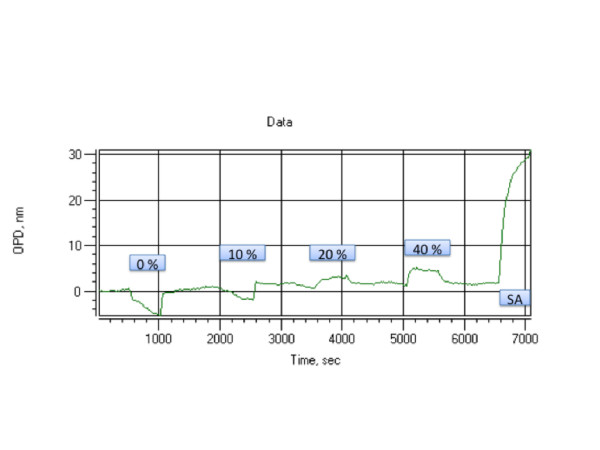
Non-specific binding properties of benzaldehyde-functionalized nanoporous silicon surface. Increasing concentrations of rat plasma, diluted in PBS buffer with 0.05 g/L citrate, were applied to a chip, using PBS as reference. Application of 10% (v/v) diluted plasma does not lead to an appreciatable amount of binding, while 20% (v/v) and 40% (v/v) rat plasma lead to a temporary signal during sample application. After exposure to complex plasma mixtures, the chip surface remains competent for specific binding with HyNic-activated streptavidin (SA). The negative signal in 0% (v/v) and 10% (v/v) rat plasma is likely due to buffer effects. 50 μL sample and reference volumes each were exposed to the sensor surface over 500 seconds.

Based on our observations above, the OPD shift represents a measure of protein interaction with the surface and is a valid approach that accurately describes the kinetics of the reaction. To test the compatibility of the platform with solvents typically used in screening of small molecule libraries, we tested the effect of 5% dimethylsulfoxide (DMSO) and 2% dimethylformamide (DMF) on the OPD-shift reading (data not shown). DMSO and DMF had no effect on the binding of our reference proteins suggesting that accurate measurements of interaction can be accomplished in the presence of compounds solubilized in these common organic solvents [15].

Many research and clinical studies require the analysis of individual proteins that are part of a complex mixture of proteins, such as plasma, cerebrospinal fluid, urine, and saliva. Key in these applications is to minimize non-specific interactions of random proteins with the surface, which could interfere with the specificity of binding and thus authenticity of results.

Typically for an experiment, we first ensured that the sample and reference flow cells are identical. Figure 6a shows that indeed sample and reference cells are identical in terms of being able to immobilize equivalent amounts of HyNic functionalized streptavidin (HyNicSA) to a benzaldehyde surface. Using the same flow cell with a new chip, we further demonstrate that there is no appreciable difference in either rate or absolute quantity of HyNicSA binding to the surface, suggesting that the aromatic hydrazone benzaldehyde reaction is not affected by the presence of other proteins (Figure 7a). These surfaces are equally competent in being able to bind biotinylated substrates (Figure 7b). We then exposed a streptavidin surface to either biotinylated BSA (bBSA) dissolved in 10% (v/v) delipidated rat plasma (sample), or dissolved in PBS, pH 7.2 (reference). As shown in figure 7b, there is no significant difference in either rate or quantity of biotinylated BSA binding when the protein is recruited from buffer, or from a complex protein mixture such at rat plasma, corresponding to a 50 fold excess protein mass. Similarly, 10% (v/v) fetal calf serum supplemented modified Eagles medium, does not impede the recruitment of bBSA when compared to bBSA binding alone (Figure 7c). This holds until much higher concentration of sera is utilized (typically 25% (v/v) for the proteins tested).

**Figure 6:**
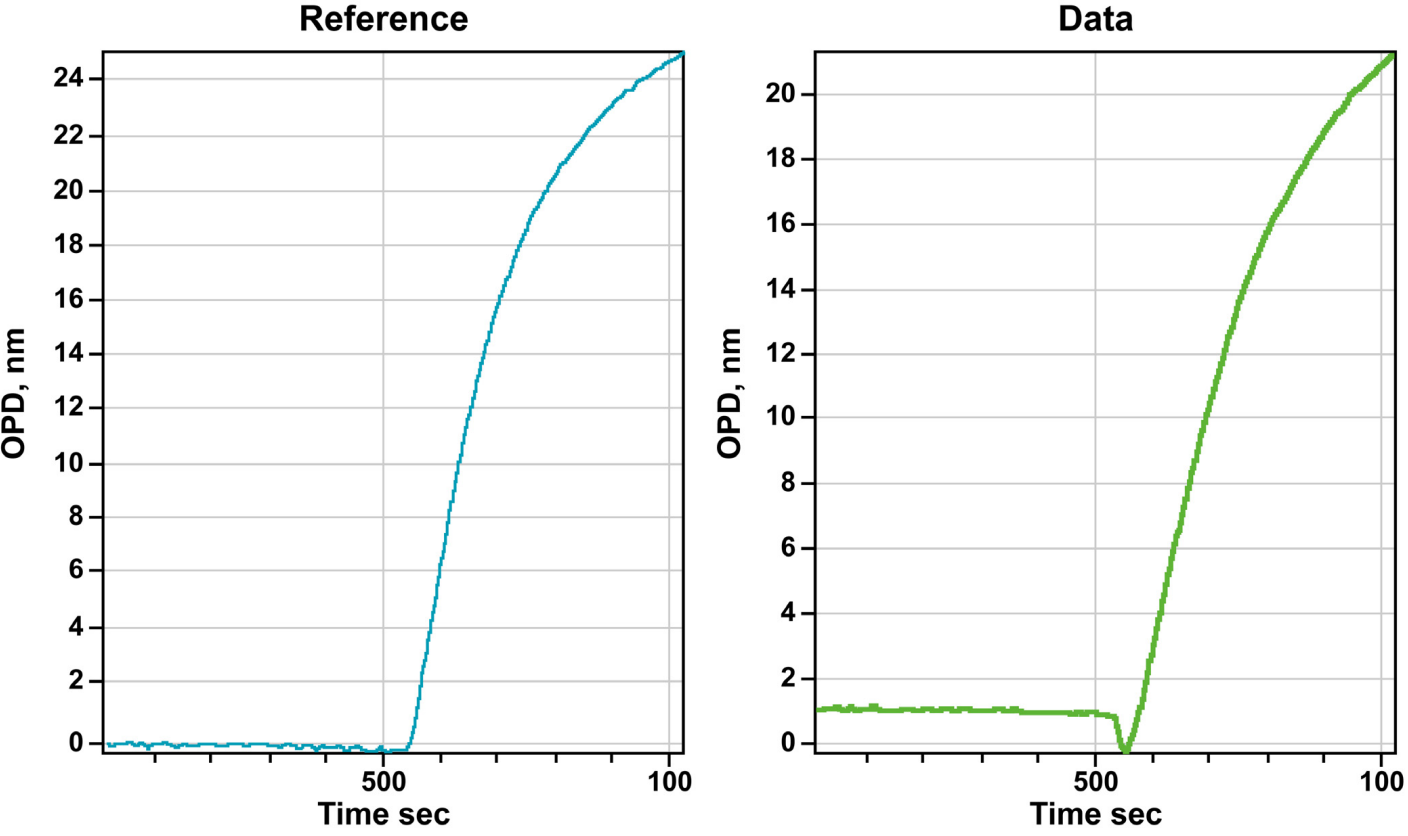
Sample (green) and reference (blue) cells behave identically during HyNic-functionalized deposition of 0.5 mg/mL HyNic functionalized streptavidin, indicating that sample flow and deposition chemistries occur at similar rate. 50 μL sample and reference volumes each were exposed to the sensor surface over 500 seconds.

**Figure 7:**
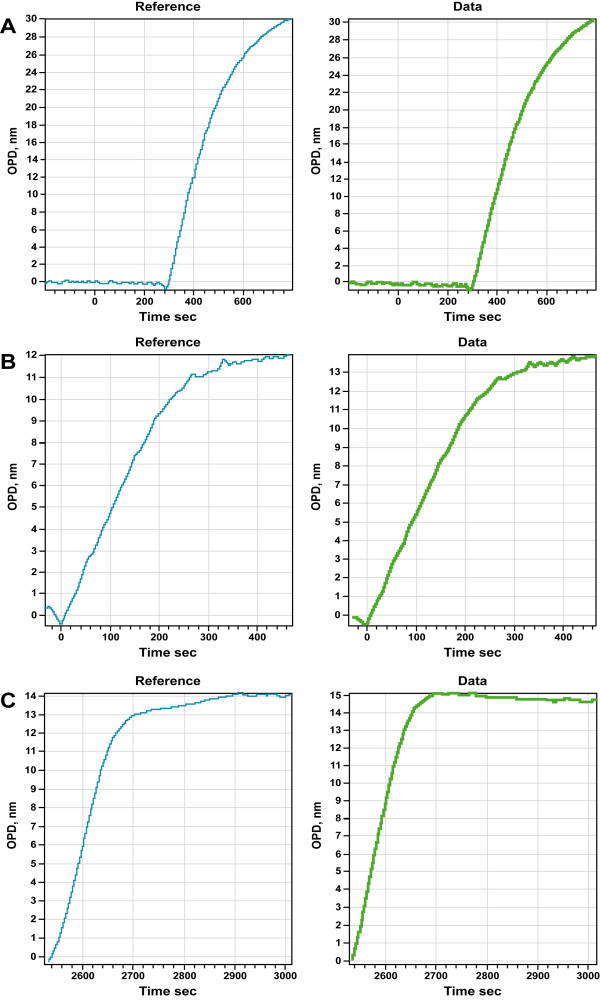
**A) **Immobilization of 0.5 mg/mL HyNic-functionalized streptavidin from PBS buffer (blue) and from 10% (v/v) rat plasma diluted in PBS (green). **B) **Immobilization of 0.1 mg/ml biotinylated BSA from PBS buffer (blue) and from 10% (v/v) rat plasma diluted in PBS (green) onto a 40% saturated streptavidin surface. **C) **Immobilization of 0.1 mg/ml biotinylated BSA from PBS buffer (blue) and from DIMEM medium supplemented with 5% (v/v) fetal bovine calf serum (green) onto a 40% saturated streptavidin surface. 50 μL sample and reference volumes each were exposed to the sensor surface over 500 seconds.

We primarily focused on a strong protein-biotin interaction to evaluate the stability of the surface. However, most kinetic experiments on this and other platforms include weaker protein-protein interactions. We therefore extended our analysis to other common protein-protein interaction pairs, such as antibody antigen interactions. We established that just like streptavidin, HyNic-functionalized human immunoglobulin can be immobilized to a benzaldehyde surface and that anti human IgG binds to this surface (Figure 8).

**Figure 8:**
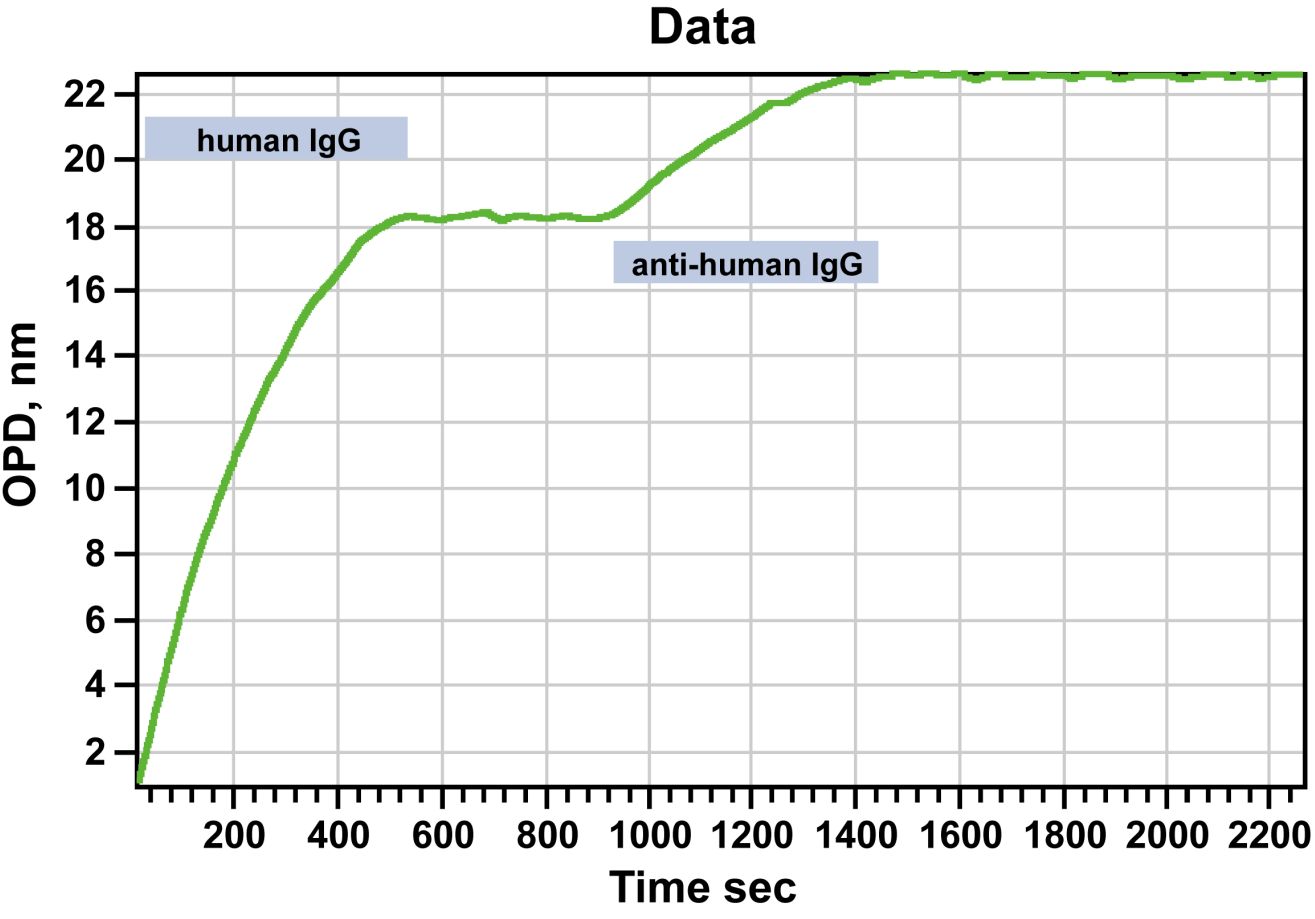
Typical full immobilization and binding run using a benzaldehyde biochip and hydrazone cross-linking chemistry. The signal shown is the result of subtracting the sample channel from the reference from the differential flow cells used with Ski Pro. 50 μL sample and reference volumes each were exposed to the sensor surface over 500 seconds. We applied 0.2 mg/ml HyNic-labeled human immunoglobulin in the sample channel and conjugation buffer in the reference channel for 500 seconds, followed by 0.1 mg/mL anti human IgG.

To directly compare the limit of detection of the nanoporous silicon interferometry based platform to a traditional SPR platform, we used identical batches of proteins to immobilize and ligands to be recruited to the surface. A typical nanoporous silicon interferometry experiment shows that human IgG/anti-human IgG can be detected at 100 ng/mL (Figure 9a), while on a SPR instrument the detection is slightly less sensitive at 1 μg/mL (Figure 9b). Dose response at the binding maximum confirms these original data (Figure 9c).

**Figure 9:**
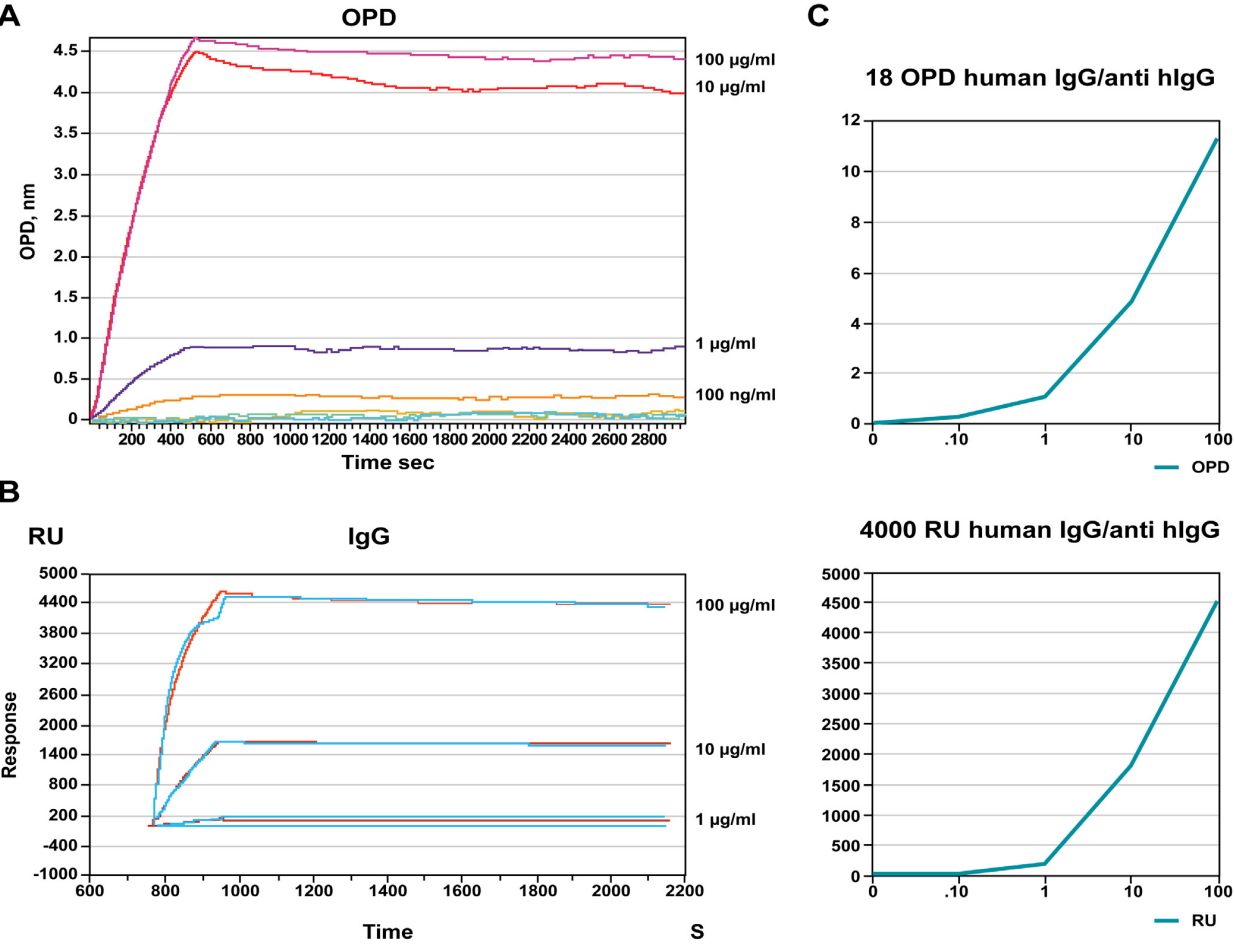
Comparison of SKiPro and Biacore 3000, using EDC/S-NHS chemistry. An 18 OPD nanoporous silicon carboxyl chip (SKi Pro) and a 4000 RU CM-5 chip (Biacore) were prepared by immobilizing human IgG from a 0.1 mg/mL solution, using identical EDV/S-NHS immobilization conditions in concordance with each manufacturer's protocols. Anti-human IgG was applied at 0, 100 ng/mL, 1 μg/mL, 10 μg/mL, and 100 μg/mL in ascending order. Each chip was regenerated by incubation with 25 mM glycine buffer, pH 2.0 for 20 minutes between exposures. Kinetic traces were displayed with SKi Pro **A) **or Biacore 3000's proprietary software **B)**. Concentration response curves were drawn in OPD (SKi Pro) and RU (Biacore) agains the concentration in μg/mL. Limit of detection for this particular protein pair is 100 ng/mL on the SKi Pro, and 1 μg/mL on the Biacore 3000 platform **C)**.

To test the influence of complex samples on the kinetics of interaction between human IgG and anti-human IgG, we calculated K_D _and on-rates for the interactions in the presence and absence of 10% delipidated rat plasma, using a fixed surface density of 18 OPD and 4000 RU, respectively, and varying anti-human IgG at 0, 0.1, 1, 10 and 100 μg/mL (Figure 10). While the on-rates are comparable between the two platforms, the presence of plasma seems to alter the on rate on the SPR platform but not on the nanoporous silicon interferometry platform. The K_D _values on the nanoporous silicon interferometry platform are consistently lower and not as much subject to interference of complex mixtures when compared to a SPR detection method, which may be caused by differences and accessibility of surface bound receptors.

**Figure 10:**
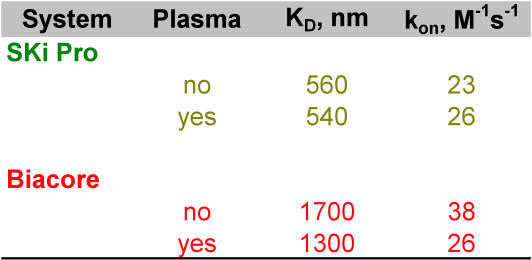
Kinetics of human IgG and anti human IgG interaction measured by nanoporous silicon interferometry and surface plasmon resonance (SPR). Kinetics of 0, 0.1, 1, 10 and 100 μg/mL anti-human IgG were measured using each platforms proprietary software and are tabulated.

## Discussion

We demonstrate the utility and flexibility of a detection method that combines a porous silicon substrate with white-light optical interferometry to measure protein-protein interactions taking place at the surface. Using either a well understood EDC/S-NHS ester immobilization chemistry to attach a receptor to the surface, or a benzaldehyde surface immobilization chemistry that involves functionalizing the receptor in solution though incorporation of hydrazine label, we exemplify how such a biosensor can be used to measure protein-protein interactions. The latter approach enables to efficiently and reproducibly generate receptor surfaces for kinetic and high-throughput applications by minimizing sample-to sample or chip-to-chip variability. This approach also allows to establish protein functionalization conditions coupled to activity based enrichment methods (e.g. affinity purification of antibodies) to ensure that the immobilized receptor retains biological function, prior to immobilization on the porous silicon surface.

A comparison of specific protein recruitment from buffer or from a complex mixture of proteins demonstrates that the porous silicon chips are able to specifically recruit proteins to either a chemical or to a protein affinity surface. It is now possible to either use hydrazone-functionalized proteins, or biotinylated proteins, or similar affinity reagents to recruit proteins and protein complexes from relevant biological protein mixtures.

The combination of sensitivity with very low non-specific binding enable this platform to be used directly on complex samples. For example, in the field of protein diagnostics, testing for autoimmune diseases could be readily adapted to this platform by providing antigens and determining the presence of antibodies in the sera of patients. Severity of the disease could be a function of the breadth of reactivity against a greater number of self-antigen, higher affinity or amount of circulating antibodies. Similarly, allergen testing could be performed by immobilizing allergens on a biochip and screening for the presence of antibodies in patients that bind to the allergen. Conceptually, the instrument platform could test for small molecules with therapeutic potential in an indirect fashion. The approach would be to allow interaction of two proteins of interest and introduce the small molecule compound(s) and determine if the compound modulates the original protein-protein interaction.

## Methods

### Reagents and Supplies

Unless otherwise mentioned, all chemicals and reagents were obtained from Sigma-Aldrich (St. Louis, MO). Succinimidyl 6-hydrazinonicotinate acetone hydrazone (S-HyNic; S1002), 10 × modification buffer, 10 × conjugation buffer, and DMF were purchased from Silicon Kinetics (San Diego, CA). Carboxy-functionalized and benzaldehyde functionalized nanoporous silicon biochips were from Silicon Kinetics (San Diego, CA). Immuno-pure streptavidin (21125), consumables, and plastic ware were supplied by Thermo Fisher (Waltham, MA). ZEBA columns were from Pierce, now Thermo Fisher (Waltham, MA).

### Surface Immobilization

To produce hydrazine-functionalized streptavidin, 5 mg of lyophilized streptavidin was dissolved in 0.5 mL of water. The dissolved streptavidin was equilibrated into PBS buffer, pH 7.2, using ZEBA columns. 1 mg S-HyNic was dissolved in 0.03 mL anhydrous DMF. After complete solubilization of the S-HyNic reagent, 15 μL of the reagent was added to the dissolved protein, followed by immediate rapid vortexing. After incubation of the labeling reaction at room temperature for 4 hours, unincorporated S-HyNic reagent was removed and buffer exchange into PBS, pH 6.0, performed using a ZEBA column. If needed, the protein concentration of the protein solution was adjusted, and aliquots of the now hydrazone-functionalized protein were frozen and stored at -20C, or directly processed. 50 μL of a 1 mg/mL HyNic-streptavidin solution in PBS, pH 6.0, was applied to a benzaldehyde nano-porous silicon chip at a flow rate of 10 μL/min, followed by an exchange into PBS, pH 7.2. 50 μL of a 200 μg/mL biotinylated BSA solution was applied at a flow rate of 10 μL/min, followed by a wash step and application of 50 μL of a 10% v/v rat plasma solution diluted into PBS buffer, pH 7.2 to test for non-specific binding.

Alternatively, 50 μL of a 1 mg/mL HyNic-streptavidin solution in PBS, pH 6.0, was applied as a comparison (reference). In a separate experiment 50 μL of a 200 μg/mL biotinylated BSA solution in 10% delipidated rat plasma (sample), or in PBS buffer, pH 7.2 (reference), was applied at a flow rate of 10 μL/min, followed by a buffer step to test for dissociation.

### Carboxyl-chip immobilization

To immobilize human immunoglobulin G (IgG) onto nanoporous silicon (SKi Pro) or a CM-5 gold surface (Biacore), we followed the manufacturers guidelines for immobilization. In brief, surfaces were activated with EDC/S-NHS, and 100 μg/ml human IgG was exposed either 5 (SKi Pro) or 3 minutes (Biacore 3000) to the activated surface. The surface was then blocked with 1 M ethanoleamine solution for 20 minutes and equilibrated into PBS running buffer. A reference surface was prepared by activating and subsequently blocking the surface under identical conditions.

### Data Acquisition and Analysis

All data obtained in this study were acquired on a Silicon Kinetics Ski Pro system, and data were analyzed using a beta version of the SKi Report software. Raw data were smoothed through averaging every ten data points and zeroed using the appropriate function keys. Biacore data were obtained on a Biacore 3000 system and analyzed using instrument-specific software.

## Competing interests

The authors declare receiving financial assistance from Silicon Kinetics for performing some of the experiments presented in the manuscript. JC is on the Scientific Advisory board of Silicon Kinetics.

## Authors' contributions

ML has carried out the majority of experiments conducted in this study. JC has conceived major aspects of the study, and participated in its design and execution. All authors read and approved the final manuscript.
